# 17DD and 17D-213/77 Yellow Fever Substrains Trigger a Balanced Cytokine Profile in Primary Vaccinated Children

**DOI:** 10.1371/journal.pone.0049828

**Published:** 2012-12-10

**Authors:** Ana Carolina Campi-Azevedo, Luiza Pacheco de Araújo-Porto, Maria Luiza-Silva, Maurício Azevedo Batista, Marina Angela Martins, Renato Sathler-Avelar, Denise da Silveira-Lemos, Luiz Antonio Bastos Camacho, Reinaldo de Menezes Martins, Maria de Lourdes de Sousa Maia, Roberto Henrique Guedes Farias, Marcos da Silva Freire, Ricardo Galler, Akira Homma, José Geraldo Leite Ribeiro, Jandira Aparecida Campos Lemos, Maria Auxiliadora-Martins, Iramaya Rodrigues Caldas, Silvana Maria Elói-Santos, Andréa Teixeira-Carvalho, Olindo Assis Martins-Filho

**Affiliations:** 1 Laboratório de Biomarcadores de Diagnóstico e Monitoração, Centro de Pesquisas René Rachou, FIOCRUZ, Belo Horizonte, Minas Gerais, Brasil; 2 Núcleo de Pesquisas em Ciências Biológicas, Universidade Federal de Ouro Preto, Ouro Preto, Minas Gerais, Brasil; 3 Escola Nacional de Saúde Pública - FIOCRUZ, Rio de Janeiro, Rio de Janeiro, Brasil; 4 Instituto de Tecnologia em Imunobiológicos, Bio-Manguinhos - FIOCRUZ - Rio de Janeiro, Rio de Janeiro, Brasil; 5 Secretaria de Estado de Saúde, Governo do Estado de Minas Gerais, Belo Horizonte, Minas Gerais, Brasil; 6 Hospital das Clínicas, Faculdade de Medicina de Ribeirão Preto, Universidade de São Paulo-USP, Ribeirão Preto, São Paulo, Brasil; 7 Diretoria Regional de Brasília, FIOCRUZ, Brasília, Distrito Federal, Brasil; 8 Departamento de Propedêutica Complementar, Faculdade de Medicina, Universidade Federal de Minas Gerais, Belo Horizonte, Minas Gerais, Brasil; Centro de Investigacion y de Estudios Avanzados del Instituto Politecnico Nacional, Mexico

## Abstract

**Background:**

This study aimed to compare the cytokine-mediated immune response in children submitted to primary vaccination with the YF-17D-213/77 or YF-17DD yellow fever (YF) substrains.

**Methods:**

A non-probabilistic sample of eighty healthy primary vaccinated (PV) children was selected on the basis of their previously known humoral immune response to the YF vaccines. The selected children were categorized according to their YF-neutralizing antibody titers (PRNT) and referred to as seroconverters (PV-PRNT^+^) or nonseroconverters (PV-PRNT^−^). Following revaccination with the YF-17DD, the PV-PRNT^−^ children (YF-17D-213/77 and YF-17DD groups) seroconverted and were referred as RV-PRNT^+^. The cytokine-mediated immune response was investigated after short-term *in vitro* cultures of whole blood samples. The results are expressed as frequency of high cytokine producers, taking the global median of the cytokine index (YF-Ag/control) as the cut-off.

**Results:**

The YF-17D-213/77 and the YF-17DD substrains triggered a balanced overall inflammatory/regulatory cytokine pattern in PV-PRNT^+^, with a slight predominance of IL-12 in YF-17DD vaccinees and a modest prevalence of IL-10 in YF-17D-213/77. Prominent frequency of neutrophil-derived TNF-α and neutrophils and monocyte-producing IL-12 were the major features of PV-PRNT^+^ in the YF-17DD, whereas relevant inflammatory response, mediated by IL-12^+^CD8^+^ T cells, was the hallmark of the YF-17D-213/77 vaccinees. Both substrains were able to elicit particular but relevant inflammatory events, regardless of the anti-YF PRNT antibody levels. PV-PRNT^−^ children belonging to the YF-17DD arm presented gaps in the inflammatory cytokine signature, especially in terms of the innate immunity, whereas in the YF-17D-213/77 arm the most relevant gap was the deficiency of IL-12-producing CD8^+^T cells. Revaccination with YF-17DD prompted a balanced cytokine profile in YF-17DD nonresponders and a robust inflammatory profile in YF-17D-213/77 nonresponders.

**Conclusion:**

Our findings demonstrated that, just like the YF-17DD reference vaccine, the YF-17D-213/77 seed lot induced a mixed pattern of inflammatory and regulatory cytokines, supporting its universal use for immunization.

## Introduction

Yellow fever (YF) vaccines have been available since the 1930s and constitute the most important method of disease control [1]. The 17D and 17DD substrains of the YF vaccine have been recommended by the World Health Organization (WHO) [2]. These substrains present minor differences in their nucleotide sequences and are considered to be safe and immunogenic [2]–[5].

Between 1938 and 1941, some weakly immunogenic vaccine substrains were identified after a varying number of passages, and some substrains exhibited greater neurovirulence contamination. The seed lot system was designed in 1942, so as to reduce the variability of vaccine lot production. In this system, a large lot of virus is produced and extensively verified for titer, sterility, and viral attenuation [6].

Bio-Manguinhos-Fundação Oswaldo Cruz is a WHO-prequalified manufacturer linked to the Brazilian Ministry of Health. It supplies the 17DD substrain YF vaccine to Brazil and other countries in South America and Africa. Because supplies may be insufficient in an emergency, comparative safety and immunogenicity studies of other YF vaccines substrains constitute a relevant challenge.

In the 1970s, a seed lot free of contaminants was developed from the YF-17D-204 substrain and maintained as stock reference (designated WHO-YF-17D-213/77), and it has been available to new manufacturers since 1977 [7]. The whole history of seed lots that have been used for production at Bio-Manguinhos since 1940 is well documented [8]. The last production seed lot, named 102/84 at passage level 285, corresponds to the YF-17DD vaccine strain and is currently available for immunization programs in Brazil. The National Immunization Program recommends YF vaccination of every Brazilian child aged between 6 and 9 months as well as the residents of endemic regions or transition areas, and a booster must be received after 10 years [9]. Vaccination is also recommended for those traveling to areas at risk of YF [10], which have expanded due to a recent YF outbreak in southeastern Brazil [11]. Because the demand for YF vaccination has increased over the last decade, the working seed lot will soon be used up and an alternative to maintain ongoing vaccine production is to use a new seed lot. According to the WHO guidelines, the following criteria must be met for approval of a new YF vaccine seed lot: i) self-limited viremia for viscerotropism analysis; ii) development of specific anti-17D antibodies by neutralization test in at least 90% of the experimental vaccinees during the immunogenicity assay; and iii) clinical score for the test virus equal to or lower than that achieved for the reference virus in terms of reactogenicity, besides a satisfactory histological score measured by appropriate tests [6].

The immunogenicity and reactogenicity of the YF-17D-213/77 substrain have been demonstrated to be equivalent to those of YF-17DD in a placebo-controlled double-blind randomized trial [12], [13]–[15]. However, despite all efforts to characterize the immune response elicited by YF vaccines, the cellular and molecular events triggered by distinct YF vaccine substrains have not been compared yet. Therefore, the major goal of the present investigation is to conduct a pioneer investigation to characterize the cytokine-mediated immune response in children submitted to primary vaccination with the YF-17D-213/77 or YF-17DD substrains and provide additional criteria to be met during the approval of a new YF vaccine seed lot. We intended to verify whether the similar cytokine-mediated immune response is linked to anti-YF humoral profile triggered by the YF-17D-213/77 or YF-17DD substrains, upon primary vaccination. We have also investigated the cytokine microenvironment elicited by the revaccination of nonresponders at primary vaccination and the association between titers of anti-YF neutralizing antibodies and the overall cytokine profile.

## Methods

### Ethics Statement

This study was approved by the Ethical Committee of Centro de Pesquisas René Rachou (CPqRR), Oswaldo Cruz Foundation – FIOCRUZ (protocol number CPqRR 21/2010), CEP-FIOCRUZ (protocol number 236A/03), SISNEP/CAAE (protocol number: 0038.1.011.000-03), CEP-SES-DF (protocol number 069/2005), and the WHO international clinical trial registration platform under the protocol number ISRCTN72367932. The complete clinical trial performed for evaluation of the reactogenicity and the immunogenicity of both vaccines has been previously published [14], [15]. The children were formally included in the study only if the free and informed consent form was signed by their parents or legal guardians.

### Study Population

**Figure 1:**
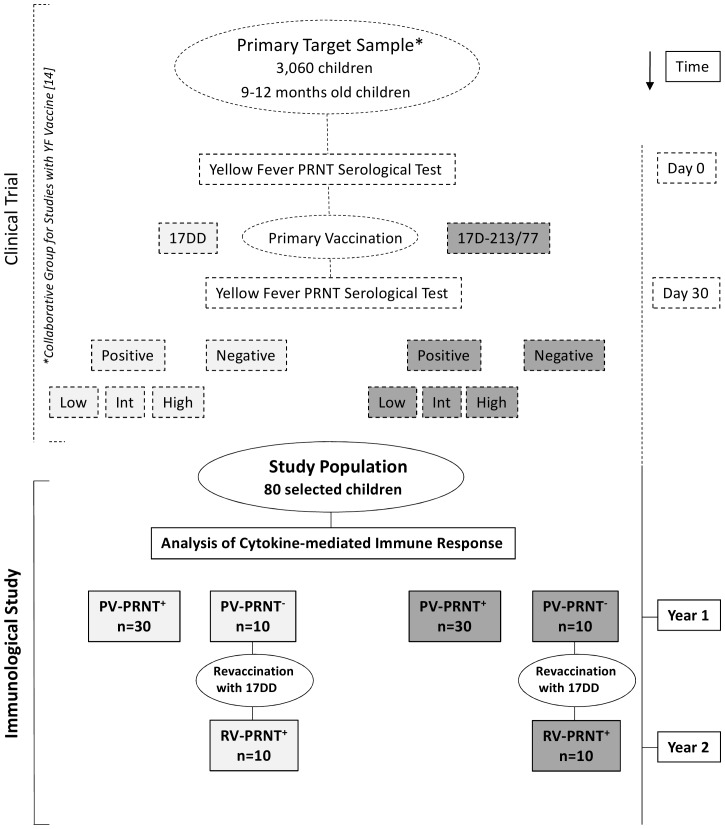
Flowchart of selection and follow-up of the Study Population from the Primary Target Sample. A total of 3,060 children, 9–12 months-old were elected for an epidemiological studies reported elsewhere [14] and referred as “Primary Target Sample”. The Clinical Trial design is highlighted by dashed format. The study population enrolled in the present investigation was selected from the “Primary Target Sample” according to the PRNT results and comprise 30 PV-PRNT^+^ and 10 PV-PRNT^−^ individuals on each experimental arm (17DD and 17D-213/77), reaching a total of 80 volunteers. The current Immunological Study design is highlighted by solid format.

The present immunological study is an additional investigation linked to a major clinical trial performed by the Collaborative Group for Studies of Yellow Fever Vaccine aiming to compared the reactogenicity and immunogenicity of two YF-vaccine substrains, 17DD and 17D-213/77 [14] and included 80 healthy children out of the “Primary Target Sample” that comprised 3,060 children, aged between 9 and 12 months, living in the metropolitan area of Belo Horizonte (n = 519) and Juiz de Fora (n = 818), Minas Gerais and Brasília (n = 1,723), Distrito Federal, Brazil. These volunteers were elected for an epidemiological studies reported elsewhere [14]. Eligibility criteria for inclusion and exclusion in the study followed the recommendations of the Brazilian National Immunization Program for routine YF vaccination is provide elsewhere [14]. A flowchart of selection and follow-up of the study population from the “Primary Target Sample” is presented in the Figure 1.

The 17DD substrain vaccine used in the present investigation was produced from the seed lot 993FB013Z, and the WHO 17D-213/77 substrain vaccine was obtained from the seed batch of the WHO available to new manufacturers since 1977 [7]. These two substrains were utilized in a randomized placebo-controlled study defined as the YF-17DD and YF-17D-213/77 groups of volunteers, referred to as the “experimental arms” [14]. Thirty days after vaccination, children provided a 3mL sample of whole blood for quantification of the anti-YF neutralizing antibodies titers, determined by the plaque reduction neutralization test (PRNT) performed as described by Stefano et al. [16]. The PRNT tests were carried out at Laboratório de Tecnologia Virológica, Bio-Manguinhos/FIOCRUZ, under the supervision of one of the researchers (M.S.F.). A PRNT value of 2.5 log_10_ mIU/mL served as the cut-off for discrimination between seroconverters (PRNT^+^≥2.5 log_10_ mIU/mL) and nonseroconverters (PRNT^−^ <2.5 log_10_ mIU/mL). The PRNT levels measured prior to vaccination showed that the children did not have previous YF antibodies. The PRNT cut-off was defined for each experimental batch based on the reactivity of the levels of PRNT observed for the internal control (mean±2SD) of un-vaccinated individuals.

The study was based on a non probabilistic sample of volunteers selected on the basis of their humoral immune response to the vaccine, aiming at clarifying the patterns of cellular immune response. For this purpose additional blood samples were collected from a selected group of children (80 out of 3,060) according to the previously known levels of neutralizing antibodies, and included 60 from Belo Horizonte, Minas Gerais and 20 from Brasília, Distrito Federal, Brazil, whose parents gave written consent. Since the additional whole blood sample was collected from the same volunteer who already donated 3 mL of whole blood for PRNT serological test, we were not able to include a larger number of children on each experimental sub-group. Mainly due to ethical restriction to work with 9–12 month-old children, we have to decide to work with the minimal sample size required for immunological analysis (n = 10) reaching a total of 100 blood collections (40 volunteers on each experimental arm plus 10 volunteers to compose each revaccinated groups).

The study design was performed under block-blind conditions, using alphanumerical sample labeling. Sequential numbers were assigned to each volunteer according to the order of arrival of the specimens, within experimental groups (“A” and “B”). The tag A or B was necessary to guide the antigen specific *in vitro* stimulation of whole blood samples, while disguising the type of vaccine received by the volunteer. Moreover, the sequential numbers precluded the disclosure of the neutralizing antibody response. Based on the PRNT results, an independent physician at the Coordinating Study Center created a randomization list of volunteers to reach a total of 40 primary vaccinated children (PV), including 30 PV-PRNT^+^ children and 10 PV-PRNT^−^ children from each experimental arm. The YF-17DD arm was comprised of 30 PV-PRNT^+^ children (15 boys and 15 girls) and 10 PV-PRNT^−^ children (6 boys and 4 girls). The YF-17D-213/77 arm also consisted of 30 PV-PRNT^+^ children (18 boys and 12 girls) and 10 PV-PRNT^−^ children (6 boys and 4 girls). The selected volunteers remained with their original numerical code and received, by an independent physician, an additional alpha tag to identify the vaccine used at primary vaccination (“A” for 17DD and “B” for the 17D-213/77). This alpha tag was also applied to the vaccine vials used for *in vitro* specific antigenic stimulation of whole blood cultures. The codes were only opened after the conclusion of all laboratorial analysis, during data entry for data analysis. The PV-PRNT^+^ children from each vaccination arm, were initially categorized into three subgroups of ten volunteers each as they present “*low*”, “*intermediate*” or “*high*” titers of PRNT antibodies. During analysis the “*low*” and “*intermediate*” were referred to as PV-PRNT^MEDIUM+^ (2.5≤PRNT≤3.5 log_10_ mIU/mL) and the “*high*” referred to as PV-PRNT^HIGH+^ (PRNT >3.5 log_10_ mIU/mL).

One year after the primary vaccination, the PV-PRNT^−^ children from both primary vaccination arms were submitted to revaccination with the YF-17DD reference vaccine as defined in the original research protocol registered at the WHO international clinical trial platform under the number ISRCTN72367932 and also presented in the flowcharts of recruiting and follow-up of study subjects described elsewhere by the Collaborative Group for Studies with Yellow Fever Vaccine [14]. The protocol informed that all volunteers presenting “NEGATIVE” YF (PRNT) serological test after primary vaccination should be submitted to revaccination. Although, according to Brazilian Healthy Ministry policy, the YF-revaccination of children and adults should be performed after 10 years, due to ethical reasons, once identified as seronegative primary vaccinees, the volunteers must be submitted to revaccination and to a complementary PRNT serological test to confirm seroconversion. From the PV-PRNT^−^ children, we have selected 10 from each experimental arm, to be included in the present investigation. All PV-PRNT^−^ children seroconverted after revaccination and were designated RV-PRNT^+^. One year after primary vaccination or revaccination, coded peripheral blood samples (7 mL) were collected into Vacationer tubes containing sodium heparin (Becton Dickinson, San Jose, CA). These coded samples were used for *in vitro* short-term whole blood cultures and intracytoplasmic cytokine analyses by flow cytometry.

### Immunophenotyping and intracytoplasmic cytokine analysis by flow cytometry

Short-term *in vitro* cultures of whole blood samples were performed as described by Luiza-Silva et al. [17] and modified as follows: aliquots of heparinized peripheral blood were incubated with RPMI 1640 medium (GIBCO, Grand Island, NY; control culture), live attenuated YF-17DD vaccine (lot 055VFA054P, Bio-Manguinhos, FIOCRUZ), or the YF-17D-213/77 seed lot (lot 05475VFA, Bio-Manguinhos, FIOCRUZ) for 6 hours at 37°C and 5% CO_2_. YF-17DD and YF-17D-213/77 were used at a final concentration of 2.5×10^3^ Plaque Forming Units – PFU/mL (approximately 450 mouse lethal dose - MLD_50_) in RPMI 1640. After *in vitro* YF-17DD or YF-17D-213/77 antigen priming, the cells were reinsulated with Brefeldin A (BFA; Sigma Chemical Company, St. Louis, MO) at 10 µg/mL for an additional period of 4 hours at 37°C and 5% CO_2_, which was followed by treatment with ethylenediamine tetraacetic acid (EDTA; Sigma Chemical Company) at a final concentration of 2 mM for 10 minutes at room temperature. The cell suspension was washed with fluorescence-activated cell sorting (FACS) buffer (phosphate-buffered saline [PBS], pH 7.2, supplemented with 0.5% bovine serum albumin and 0.1% sodium azide, all from Sigma Chemical Company), and aliquots were stained with fluorescent anti-human cell surface monoclonal antibodies (all from Caltag, Burlingame, CA): anti-CD4-TC (clone #S3.5), anti-CD8-TC (clone #3B.5), anti-CD14-TC (clone #TüK4), anti-CD16-TC (clone #3G8), and anti-CD19-TC (clone #ST25-C1) for 30 minutes at room temperature. After membrane staining, erythrocyte lysis, and leukocyte fixation, the cell suspension was permeabilized with FACS perm-buffer (FACS buffer supplemented with 0.5% saponin). Next, aliquots were incubated with fluorescent anti-cytokine monoclonal antibodies (all from BD-Pharmingen, San Jose, CA), including anti-IL-12-PE (clone #C11.5), anti-IFN-γ-PE (clone #4S.B3), anti-TNF-α-PE (clone #Mab11), anti-IL-4-PE (clone #8D4-8), anti-IL-5-PE (clone #TRFK5), and anti-IL-10-PE (clone #JES3-19F1), for 30 minutes at room temperature in the dark. After intracytoplasmic cytokine staining, the leukocytes were washed with FACS perm-buffer and FACS buffer, followed by fixation in FACS fixing solution (10 g/L paraformaldehyde, 10.2 g/L sodium cacodylate, and 6.63 g/L sodium chloride, pH 7.2).

### Flow Cytometry Acquisition and Analysis

Immunostained samples were acquired using a FACScalibur™ flow cytometer equipped with a four-color detection system (Becton Dickinson, San Jose, CA, USA); the CELLQUEST software (Franklin Lakes, NJ, USA) was employed for data acquisition and analysis. A total of 30,000 events/tube were registered and stored for further analysis. Distinct gating strategies were utilized for analysis of the cytokine-expressing leukocytes subsets, including neutrophils, monocytes, natural killer (NK) cells, and lymphocytes (CD4^+^ and CD8^+^ T-cell subsets, and B cells), as previously reported by Luiza-Silva et al. [17]. At first, the results were expressed as percentages of cytokine-positive cells for each leukocyte subpopulation.

### Overall Pattern and Cytokine Signature Analysis

The cytokine-mediated immune response elicited by the YF-17D-213/77 seed lot and the YF-17DD licensed vaccine was assessed after *in vitro* recall with specific antigenic stimulation. Analysis of the intracytoplasmic cytokine profile of peripheral blood leukocytes initially yielded the percentage of cytokine-positive cells. The cytokine index was then calculated as the ratio between the percentages of cytokine-positive cells observed in the stimulated cultures (YF-17DD or YF-17D-213/77) and the percentage of cytokine-positive cells detected in the unstimulated cultures (control).

Taking all data recorded for both experimental arms (YF-17DD and YF-17D-213/77, referred to as YF-Ag as a whole), we computed the global median of cytokine indexes (YF-Ag/control) for each leukocyte subpopulations, including neutrophil (NEU), monocyte (MON), and lymphocyte subsets. The global median of IL-12^+^NEU = 0.92; IFN-γ^+^NEU = 0.84; TNF-α^+^NEU = 1.11; IL-4^+^NEU = 0.96; IL-10^+^NEU = 0.95; IL-12^+^MON = 1.14; TNF-α^+^MON = 1.92; IL-10^+^MON  = 0.95; IFN-γ^+^NK = 1.15; TNF-α^+^NK = 1.25; IL-4^+^NK = 1.25; IL-12^+^CD4^+^ = 1.13; IFN-γ^+^CD4^+^ = 1.11; TNF-α^+^CD4^+^ = 1.46; IL-4^+^CD4^+^ = 1.51; IL-5^+^CD4^+^ = 1.32; IL-10^+^CD4^+^ = 1.32; IL-12^+^CD8^+^ = 1.11; IFN-γ^+^CD8^+^ = 1.24; TNF-α^+^CD8^+^ = 1.21; IL-4^+^CD8^+^ = 1.26; IL-5^+^CD8^+^ = 1.24; IL-10^+^CD8^+^ = 1.53; TNF-α^+^CD19^+^ = 0.71; IL-4^+^CD19^+^ = 0.79; IL-10^+^CD19^+^ = 0.75 was utilized as the cut-off, in order to segregate low, high inflammatory, or high regulatory cytokine indexes. This strategy allowed for computation of the percentage of volunteers displaying high cytokine indexes. The cytokine signature for each experimental arm was also calculated for each assembled leukocyte subset. The cytokine-mediated immune response was first addressed as the “overall cytokine pattern” of the whole leukocyte population (NEU, MON, and lymphocyte subsets). Next, a detailed analysis of the major sources of each cytokine, denominated “cytokine signatures” as previously suggested by Luiza-Silva et al. [17], was carried out. These data were assembled as the ascendant frequency of high cytokine indexes for PV-PRNT^+^ that was taken as the reference cytokine curve for the YF-17DD or the YF-17D-213/77 arm, in order to identify changes in the overall cytokine patterns observed for the other experimental arms. Each axis represents the frequency (%) of volunteers exhibiting high cytokine indexes.

### Comparative Data Analysis

**Figure 2:**
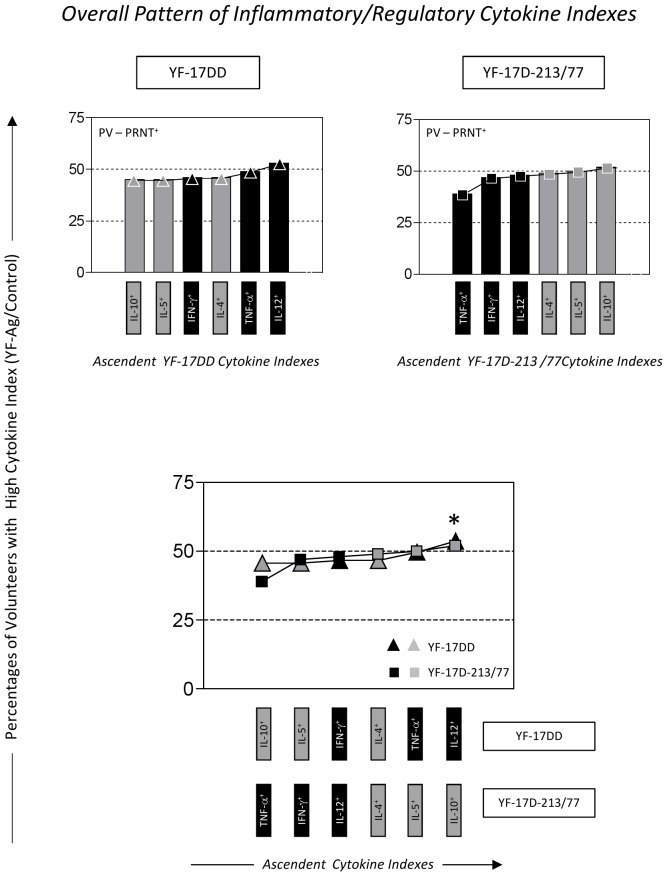
Overall inflammatory and regulatory cytokine pattern triggered by the YF-17DD and YF-17D-213/77 substrains upon the *in vitro* recall of whole blood leukocytes from seroconverter children (PV-PRNT^+^) with specific YF-Ag. The overall pattern of inflammatory (black bar) and regulatory (gray bar) cytokines is presented as the ascendant percentages of volunteers with high cytokine indexes, taking the cytokine profile of the whole leukocyte population (neutrophils, monocytes, and lymphocytes) for each experimental arm. Comparative analysis of the overall cytokine patterns from YF-17DD (lines with black or gray triangles) and YF-17D-213/77 (lines with black or gray squares) vaccines were further compared by overlapping the ascendant cytokine curves (bottom panel). Dotted lines highlight the 25^th^ and 50^th^ percentiles used as reference for comparative analysis. *Relevant differences were considered when the frequency for a given cytokine emerged outside the 50^th^ percentile as compared to the reference cytokine pattern or signature.

**Figure 3:**
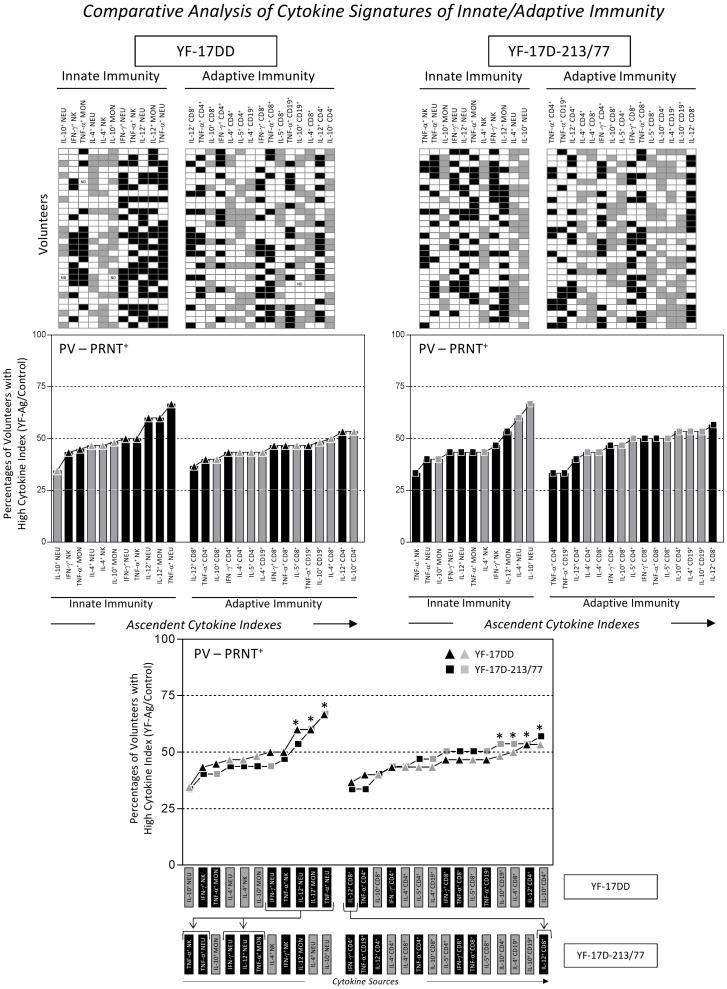
Comparative analysis of the cytokine signatures of innate and adaptive immunity triggered by the YF-17DD and YF-17D-213/77 substrains upon the *in vitro* recall of whole blood leukocytes from seroconverter children (PV-PRNT^+^) with specific YF-Ag. The diagrams highlight each leukocyte subsets with distinct tags as they display low (white rectangle) or high (black rectangle = inflammatory, gray rectangle = regulatory) cytokine producers (top panel). The ascendant frequency of volunteers with high cytokine indexes of the innate and adaptive immunity was assembled for each experimental arm and is demonstrated by bar graphs (medium panel). Comparative analysis of the overall cytokine patterns of YF-17DD (lines with black or gray triangles) and YF-17D-213/77 (lines with black or gray squares) vaccinees were further compared by overlapping the ascendant cytokine curves (bottom panel). Dotted lines highlight the 25^th^ and 50^th^ percentiles used as reference for comparative analysis. *Differences were considered relevant when the frequency for a given cytokine emerged outside the 50^th^ percentile as compared to the reference cytokine pattern or signature.

**Figure 4:**
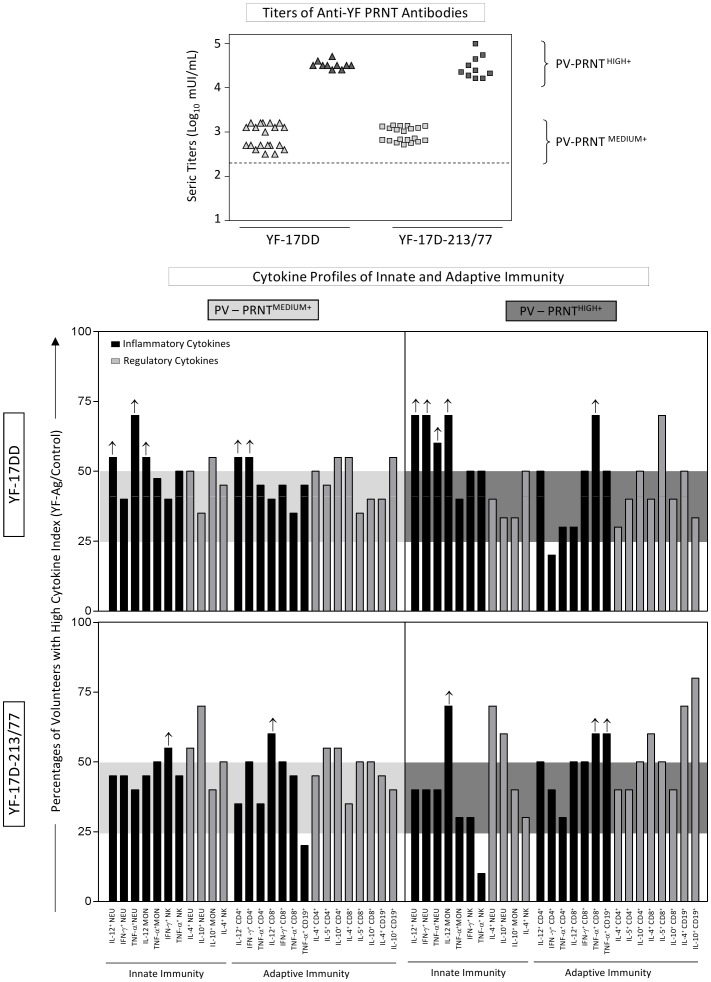
Impact of serum titers of anti-YF neutralizing antibodies on the cytokine-mediated immune response triggered by the YF-17DD and YF-17D-213/77 substrains upon *in vitro* recall of whole blood leukocytes from seroconverter children (PV-PRNT^+^) with specific YF-Ag. The PRNT^+^ groups from each experimental arm were first categorized into two subgroups referred to as PV-PRNT^MEDIUM+^ (2.5≤ serum titers ≤3.5 log_10_ mIU/mL) and PV-PRNT^HIGH+^ (serum titers >3.5 log_10_ mIU/mL). The cytokine profile of the PV-PRNT^MEDIUM+^ and PV-PRNT^HIGH+^ subgroups were evaluated, considering relevant the percentages of a given inflammatory cytokine that emerged higher than the 50^th^ percentile, as indicate by an upward arrow (↑).

**Figure 5:**
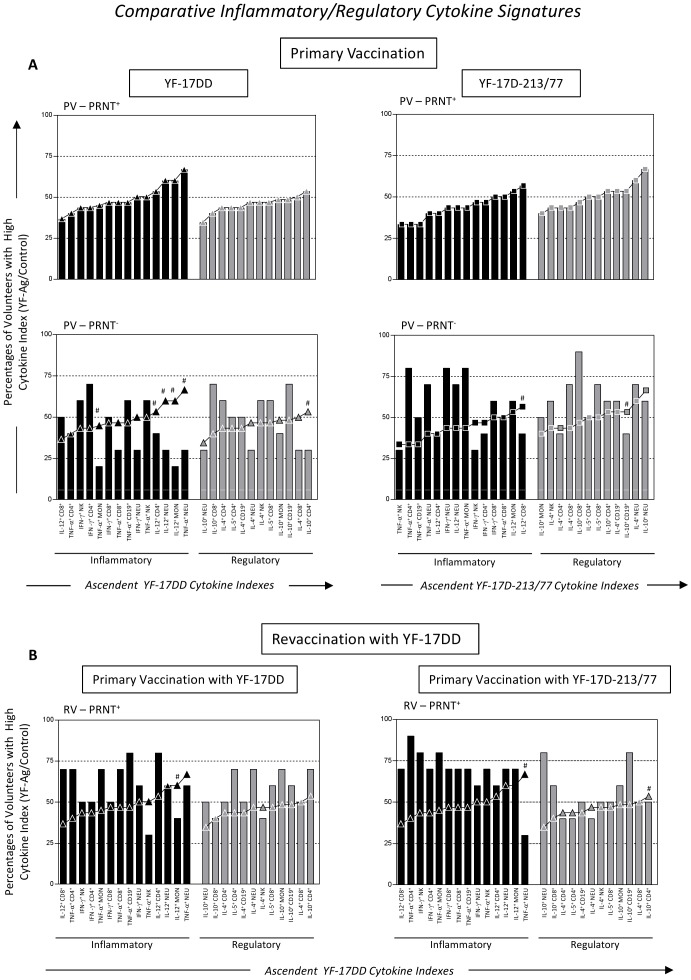
Comparative inflammatory and regulatory cytokine signatures triggered by the YF-17DD and YF-17D-213/77 substrains upon the *in vitro* recall of whole blood leukocytes from (A) seroconverter (PV-PRNT^+^) and nonseroconverter primary vaccinees (PV-PRNT^−^) as well as (B) seroconverter revaccinees (RV-PRNT^+^) with specific YF-Ag. The ascendant frequency of volunteers with high inflammatory and regulatory cytokine indexes was assembled for each experimental arm and demonstrated by bar graphs. Comparative analysis between PV-PRNT^+^, PV-PRNT^−^, and RV-PRNT^+^ within the same experimental arm was performed taking the ascendant cytokine curve of the YF-17DD (lines with black or gray triangles) or YF-17D-213/77 (lines with black or gray squares) groups as reference. #Differences were considered relevant when the percentage of a given cytokine emerged below the quartile of the reference cytokine signatures.

The cytokine patterns and signatures triggered by the YF-17DD and YF-17D-213/77 substrains were compared by overlapping the PV-PRNT^+^ ascendant profiles of each experimental arm. Relevant differences were considered when the frequency for a given cytokine emerged outside the 50^th^ percentile, compared with the cytokine pattern or signature from the other experimental arm (Figures 2 and 3). The difference between the PV-PRNT^MEDIUM+^ and PV-PRNT^HIGH+^ subgroups in terms of the cytokine-mediated immune response was analyzed by considering as relevant the percentages of a given inflammatory cytokine that emerged higher than the 50^th^ percentile (Figure 4). A comparative analysis among PV-PRNT^+^, PV-PRNT^−^, and RV-PRNT^+^ within the same experimental arm was performed by taking the ascendant cytokine curve of the YF-17DD or YF-17D-213/77 group as reference, and relevant differences were considered when the percentage of a given cytokine emerged below the quartile (25^th^ percentile) of the reference cytokine signatures (Figure 5). The use of 25^th^ and 50^th^ percentile as a limit to identify relevant differences in the cytokine signatures between groups was previously proposed by Luiza-Silva et al [17]. This approach showed to be relevant to detect subtle changes in the cytokine signatures not detectable by conventional statistical approaches. The value of this qualitative method was emphasized during data analysis, as the patterns of cytokine signature were consistent with the profile of humoral immune response in both experimental arms.

## Results

### The YF-17DD substrain and the YF-17D-213/77 seed lot trigger a balanced inflammatory and regulatory overall cytokine pattern in seroconverter children

Aiming to characterize whether the cytokine-mediated immune response elicited by the YF-17D-213/77 seed lot substrain is similar to that triggered by the YF-17DD reference vaccine, we investigated the impact of specific YF-17DD/control or YF-17D-213/77/control *in vitro* recall on the cytokine profile of peripheral blood leukocytes from primary vaccinated children. For this purpose, we first characterized the overall pattern of inflammatory and regulatory cytokines as the ascendant frequency of high cytokine producers by considering the whole leukocyte population (neutrophils, monocytes, and lymphocytes).

Data regarding the overall cytokine pattern of the whole leukocyte population demonstrated that the YF-17D-213/77 and the YF-17DD substrains triggered a balanced inflammatory and regulatory cytokine pattern in PV-PRNT^+^ children, with the frequency of high cytokine producers confined between the 25^th^ and 50^th^ percentiles. A detailed analysis of the ascendant cytokine patterns revealed that the PV-PRNT^+^ group from the YF-17DD arm was characterized by IL-10<IL-5<IFN-γ<IL-4<TNF-α<IL-12, whereas PV-PRNT^+^ vaccinees from the YF-17D-213/77 arm displayed TNF-α<IFN-γ<IL-12<IL-4<IL-5<IL-10 (Figure 2, top panel).

Analysis of the overlapping curves revealed a slight predominance of high IL-12 producers among the YF-17DD vaccinees and a modest prevalence of high IL-10 producers in the YF-17D-213/77 arm (Figure 2, bottom panel).

### The cytokine signature triggered by the YF-17DD substrain counts on a relevant involvement of inflammatory status of the innate immunity, whereas the YF-17D-213/77 substrain displays a relevant inflammatory component at the adaptive immunity

We investigated the major sources of inflammatory and regulatory cytokines underlying the immune response in YF-17DD and YF-17D-213/77 vaccinees by using the global median cytokine index (YF-Ag/control) in order to highlight each leukocyte subset with distinct tags according to whether it displayed low or high cytokine production (Figure 3, top panel). After this procedure, we calculated the frequency of volunteers with high cytokine indexes for each leukocyte subset. We then used these data for assembly of the ascendant cytokine signatures of the innate and adaptive immunity.

Analysis of the cytokine signatures of PV-PRNT^+^ children demonstrated that the YF-17DD substrain triggered a prominent involvement of inflammatory status of the innate immunity, especially due to high cytokine indexes of IL-12 in neutrophils and monocytes and of TNF-α in neutrophils. In the YF-17D-213/77 arm, the PV-PRNT^+^ presented a relevant participation of regulatory cytokines from the innate immunity, especially due to enhanced IL-4 and IL-10 indexes in neutrophils (Figure 3, middle panel). Analysis of the cytokine signature promoted by the YF-17DD in terms of adaptive immunity revealed a balanced involvement of inflammatory and regulatory cytokines from T-cell subsets and B lymphocytes with prominent participation of IL-12 and IL-10 from CD4^+^ T cells. The YF-17D-213/77 substrain triggered a mixed involvement of inflammatory and regulatory cytokines, particularly due to enhanced IL-12 index in CD8^+^ T cells (Figure 3, middle panel).

Our findings also showed that the cytokine signature of PV-PRNT^+^ triggered by the YF-17D-213/77 substrain was characterized by higher involvement of regulatory neutrophils via IL-4 and IL-10. Moreover, the cytokine signature of PV-PRNT^+^ triggered by the YF-17D-213/77 substrain with respect to adaptive immunity displayed a regulatory profile characterized by IL-10 from CD4^+^ T cells and IL-10 and IL-4 from B cells, as well as prominent participation of an inflammatory response via IL-12 from CD8^+^ T cells (Figure 3, middle panel).

Comparative analysis of cytokine signature curves highlighted the particularities of the cytokine-mediated immune response prompted by the YF-17DD *in vitro* recall, as shown by the shift of innate immunity inflammatory cytokines^+^ cells toward higher frequencies (specially IFN-γ^+^NEU, TNF-α^+^NK, IL-12^+^NEU, IL-12^+^MON, and TNF-α^+^NEU). On the other hand, we noted the contribution of inflammatory cells of adaptive immunity (IL-12^+^CD8^+^) for the YF-17D-213/77 arm (Figure 3, bottom panel).

### The serum levels of anti-YF-neutralizing antibodies are associated with distinct cytokine-mediated immune response following YF-17DD and 17D-213/77 vaccinations

Aiming to verify whether the level of anti-YF neutralizing antibodies would play a role in controlling the cytokine-mediated immune response triggered by the *in vitro* recall with YF-Ag, the PV-PRNT^+^ children from each vaccination arm were further categorized into two subgroups, namely PV-PRNT^MEDIUM+^ (2.5≤serum titers ≤3.5 log_10_ mIU/mL) and PV-PRNT^HIGH+^ (serum titers >3.5 log_10_ mIU/mL). The serum levels of both anti-YF PRNT antibodies in the PRNT^MEDIUM+^ and PRNT^HIGH+^ subgroups did not differ from those of the YF-17DD and YF-17D-213/77 seroconverters (Figure 4, top panel). However, there was a distinct association between the serum titers of anti-YF-neutralizing antibodies and the cytokine-mediated immune response in YF-17DD and YF-17D-213/77 primary vaccinees.

We observed a generally balanced inflammatory and regulatory cytokine profile of innate and adaptive immunity in the PV-PRNT^MEDIUM+^ subgroups from both experimental arms. In contrast, the PV-PRNT^HIGH+^ subgroup from the YF-17DD arm exhibited a polarized inflammatory cytokine profile especially in the case of the innate immunity, whereas the PV-PRNT^HIGH+^ subgroup from the YF-17D-213/77 arm had a predominant regulatory cytokine profile for both the innate and adaptive immune responses (Figure 4, bottom panel). It is noteworthy that, despite the overall distinct molecular mechanisms triggered by the cellular–humoral immunological interface in the YF-17-DD and YF-17D-213/77 arms, both vaccines were able to elicit particular but relevant inflammatory events regardless of the anti-YF PRNT antibody levels (Figure 5, arrows). These inflammatory events consisted of putative biomarkers of protection mechanisms that would be relevant in an eventual contact with wild YF strains.

### The lack of seroconversion upon primary vaccination with YF-17DD and YF-17D-213/77 substrains is associated with distinct gaps in the cytokine-mediated immune response

To further evaluate the cytokine-mediated immune response triggered by the YF-17DD and the YF-17D-213/77 vaccines, we generated the inflammatory and regulatory cytokine signature curves for the PV-PRNT^+^ groups of both experimental arms, namely YF-17DD and YF-17D-213/77 (Figure 5A, top panel). These cytokine signatures were used as reference curves for comparative analysis for identifying changes in the cytokine-mediated immune response in nonseroconverters primary vaccinees (PV-PRNT^−^) (Figure 5A). Data analysis demonstrated that the PV-PRNT^−^ children in the YF-17DD arm presented a relevant gap in the cytokine signature, especially in the inflammatory profile of the innate immunity. In fact, the PV-PRNT^−^ group from the YF-17DD arm had a relevant deficiency of neutrophil- and monocyte-derived IL-12 and TNF-α. In addition, they displayed considerably deficient synthesis of IL-10 and IL-12 by CD4^+^ T cells as well as of IL-4 by CD8^+^ T cells (Figure 5A).

On the other hand, the PV-PRNT^−^ children of the YF-17D-213/77 arm exhibited a relevant gap in the synthesis of inflammatory cytokines, particularly a deficiency of IL-12 produced by CD8^+^ T cells, besides the drop in the IL-10 produced by B cells as compared to the reference cytokine signature (Figure 5A).

### Revaccination with YF-17DD brings out an overall balanced cytokine profile in YF-17DD nonresponders and a robust inflammatory profile in YF-17D-213/77 nonresponders

Aiming to investigate whether revaccination would restore the cytokine-mediated immune response of nonseroconverter primary vaccinees, we re-evaluated the immunological status of the PV-PRNT^−^ groups from both experimental arms. Our findings demonstrated that revaccination with YF-17DD was able to induce seroconversion in all revaccinees (data not shown). Interestingly, revaccination with the YF-17DD substrain was able to restore most of the gaps observed in the cytokine signature, leading to an overall balanced cytokine profile in YF-17DD nonresponders (Figure 5B). In fact, revaccination restored the ability of neutrophils from RV-PRNT^+^ children belonging to the YF-17DD arm to produce TNF-α and IL-12 as well as the capacity of CD4^+^ T cells to generate IL-12 and IL-10 (Figure 5B). Similarly, the YF-17DD revaccination of nonseroconverter children from the YF-17D-213/77 arm re-established the ability of CD8^+^ cells to produce IL-12 as well as the capacity of CD19^+^ B cells to generate IL-10 (Figure 5B).

Together, these findings indicated that revaccination not only repaired the serological status but also re-established the overall balanced cytokine profile in YF-17DD nonresponders and a robust inflammatory profile in YF-17D-213/77 nonresponders to primary vaccination, filling most of the gaps observed in nonseroconverters.

## Discussion

As YF immunization increases worldwide and the demand for vaccine becomes higher, an alternative to maintain ongoing vaccine production is to use the new seed lot that can provide vaccines for immunization programs. Hence, new seed lots must be prepared and tested according to the Good Clinical Practice guidelines. These guidelines include a comparative analysis of immunogenicity and reactogenicity with the already licensed vaccine.

Aiming to further contribute to the validation of the YF-17D-213/77 and the YF-17DD substrains for use in immunization programs, in the present investigation we assessed the cellular and humoral immune response triggered by these vaccine substrains. This research was conducted as a complementary parameter under Good Clinical Practice guidelines, and it could aid national immunization programs in Brazil and other parts of Latin America and Africa with respect to clinical validation studies of a new seed lot. Moreover, analysis of these YF vaccine substrains with different passage histories should reveal the extent of immunological alterations associated with the already reported genetic variability between the YF-17D-213/77 seed lot and the YF-17DD vaccine.

First, we characterized the overall cytokine-mediated immune response to YF vaccination using a recently proposed approach for identifying the frequency of high cytokine producers amongst the YF-17D-213/77 and the YF-17DD seroconverters primary vaccinees. Our findings demonstrated that the YF-17D-213/77 and the YF-17DD substrains trigger a balanced overall inflammatory and regulatory cytokine-mediated immune response among the seroconverter primary vaccinated children. These results agree with previous reports that the induction of complex immune response, including activation and modulation events as well as a mixed and balanced pro-inflammatory and regulatory cytokine profile, is relevant to the development of a nondeleterious immune response in YF-17DD primary vaccinees [17], [18]–[23]. This would be relevant in terms of guaranteeing the establishment of protective events in an eventual contact with the wild YF virus. Insights from the past decade arising from advances in our understanding of innate and adaptive immunity following YF vaccination have provided a clearer idea that an extensive inflammatory response is deleterious to establishing immunity. Hence, it is not surprising that mechanisms exist to shut off inflammation and regulate the overall immune response following vaccination and induction of an inflammatory response [17], [18]–[24].

In this study, we observed that several cytokine sources are elicited by YF vaccination in the innate and adaptive arms of the immune response. Using flow cytometry, we demonstrated that the YF-17D-213/77 and the YF-17DD seroconverter primary vaccinees differ with respect to the “cytokine signatures” of innate and adaptive immunity. In fact, while the YF-17DD vaccine induces an important inflammatory cytokine signature in terms of innate immunity, particularly that mediated by TNF-α^+^ neutrophils and neutrophil- and monocyte-derived IL-12, which ensures the inflammatory profile in YF-17D PRNT^+^ primary vaccinees. Meanwhile, the YF-17D-213/77 experimental arm had a prominent inflammatory signature mainly represented by the outstanding adaptive immunity mediated by IL-12^+^ CD8^+^ T cells. However, it is important to mention that both substrains are able to trigger counter-balancing regulatory immune events of innate and adaptive immune response, as illustrated by the IL-10 production by CD4^+^ T cells in YF-17DD PV-PRNT^+^ subjects and the prominent frequency of IL-10^+^ neutrophils in YF-17D-213/77 PRNT^+^ primary vaccinees.

It is well recognized that the YF vaccines in current clinical use display an outstanding capacity for prompting antibody responses, particularly those that provide protection by neutralizing the YF virus [25]. The equivalent immunogenicity of YF vaccines from the 17D-213/77 and 17DD substrains has already been demonstrated by placebo-controlled double blind randomized trial [12]. Our investigation of whether the neutralizing antibody titer predicts specific cytokine-mediated immune response has shown that the serum levels of anti-YF-neutralizing antibodies are associated with distinct cytokine profiles in the YF-17DD and YF-17D-213/77 arms. However, regardless of the anti-YF PRNT antibody levels, both vaccines are able to elicit particular but relevant inflammatory events required for protection against natural infection.

It is important to bear in mind that both the cellular and humoral arms of the immune system are required for effective protection against YF natural infection [26]. Insights from innate immunity research have led to a better appreciation of how existing vaccines work and have also contributed to the rational development of new vaccines [27]. In fact, previous studies have demonstrated that the Toll-like receptors (TLR) involved in the NK-cell activation following vaccination with the YF-17DD substrain seems to play a potential role in the development of long-lasting protective memory to the YF virus [28]. Progress has been made in defining the contribution of these receptor-ligand interactions to YF-vaccine–mediated protection. Impaired cytokine production was observed when dendritic cells from TLR-deficient mice were stimulated with the 17D-204 vaccine [18].

Some of the studies that address the complex interactions between the virus and the immune system suggest that the ability of YF-vaccines to rapidly produce neutralizing PRNT antibodies as well as the CD8^+^ T cell effectors. A cardinal property of memory CD8^+^ T cells is their ability to undergo rapid proliferation upon reencountering the priming antigen. This is an important component of protective immunity; a higher proliferative potential implies a larger pool of secondary effectors. Akondy et al [29] have define the attributes of a human CD8^+^ T cell response that generates high-quality immune memory by performing a comprehensive analysis of the CD8^+^ T cells elicited after vaccination with the efficacious yellow fever live virus vaccine. The YF-specific CD8^+^ T cells displayed broad specificity, high magnitude, multiple functions, robust proliferative potential, and long-term persistence, all characteristics of protective cellular immunity.

To our knowledge, there are no systematic data on the magnitude and relevance of the antigen-specific CD8^+^ T-cell response necessary for protection against YF infection. However, evidence has shown that both the CD8^+^ T-cell and the antibody responses can be critical parameters of protective immunity upon YF-17D-204 vaccination. Using the systems biology approach, recent studies have identified relevant aspects of the innate immunity that can be used to predict both neutralizing antibody and CD8^+^ T-cell responses to YF-17D-204 vaccination [24]. In the case of the present investigation, it is noteworthy that the YF-17D-213/77 substrain, a close relative of the YF-17D-204 substrain, prompts a cytokine-mediated immune response with important contribution from CD8^+^ T cells. On the other hand, the YF-17DD (derived from the YF-17D substrain with independent passage in tissue culture prior to propagation in embryonated chicken eggs) presents a more robust involvement of the innate immune compartment. These particularities in the source of inflammatory cytokines of innate and adaptive immunity can represent events associated with the genetic variability incorporated in the YF-17D-213/77 seed lot and the YF-17DD vaccine during the passage history of the original YF Asibi strain.

The high immunogenicity of YF vaccines has been confirmed by studies in adults and children. The YF vaccine immunogenicity in adults can reach over 95% seroconversion among subjects that were previously seronegative and it probably persists for at least 35 years [30]. However, the seroconversion rates range from 77.5% to 90% in children, with distinct seroconversion rates being observed according to the age at vaccination [15], [16], [31]–[35]. Despite the great public health success of YF vaccination programs, the lack of seroconversion in some vaccinees is a problem, since these subjects are not protected against natural YF infection. Our present evaluation of the cytokine-mediated immune response of a group of nonseroconverters after YF-17D-213/77 or YF-17DD vaccination confirmed that, besides the lack of relevant titers of anti-YF neutralizing antibodies detected by PRNT, the PV-PRNT^−^ subjects from both experimental arms exhibited relevant gaps in the inflammatory cytokine response of circulating leukocytes upon antigen-specific *in vitro* recall. Interestingly, the gaps could be selectively observed in the most relevant inflammatory component induced by each YF substrain; i.e., pro-inflammatory innate immunity in the YF-17DD arm and IL-12 response of CD8^+^ T cells in the YF-17D-213 nonseroconverters. Most importantly, our findings demonstrate that these deficiencies in the cytokine-mediated immune response can be repaired upon revaccination of the subjects that were seronegative after primary vaccination. This phenomenon has been discussed in more details elsewhere [17].

In conclusion, despite the particular sources of cytokine-mediated immune response in the innate and adaptive compartments, both vaccines (YF-17DD and YF-17D-213/77) trigged a balanced cytokine signatures, thereby corroborating their already reported attenuated phenotype [6], [10]. These findings suggest that both the YF-17DD reference vaccine and the YF-17D-213/77 seed lot trigger an overall balanced cytokine-mediated immune response, which supports their universal use for immunization purposes when it comes to preventing YF outbreaks worldwide. It is important to notify that these hypotheses should be taken with the appropriate prudence considering the qualitative and observational nature of the present investigation.
